# Assessment of Influence of Magnetic Forces on Aggregation of Zero-valent Iron Nanoparticles

**DOI:** 10.1007/s11671-010-9753-4

**Published:** 2010-08-24

**Authors:** Dana Rosická, Jan Šembera

**Affiliations:** 1 Institute of Novel Technologies and Applied Informatics, Technical University of Liberec, Liberec, Czech Republic

**Keywords:** Iron nanoparticles, Aggregation, Magnetic forces, Mass transport coefficient

## Abstract

Aggregation of zero-valent nanoparticles in groundwater is influenced by several physical phenomena. The article shortly introduces preceding works in modeling of aggregation of small particles including influence of sedimentation, velocity profile of water, heat fluctuations, and surface electric charge. A brief description of inclusion of magnetic forces into the model of aggregation follows. Rate of influence of the magnetic forces on the aggregation depends on the magnitude of magnetization of the particles, radius of nanoparticles, size of the aggregates, and their concentration in the solution. Presented results show that the magnetic forces have significant influence on aggregation especially of the smallest iron particles.

## Introduction

Zero-valent iron nanoparticles (nZVI) composed of iron and its oxides are spherical particles with diameter approximately 50 nm and with a large specific surface. These particles are used for the decontamination of groundwater and soil and especially for the decontamination of organic pollutants such as halogenated hydrocarbons [1]. Nanoparticles migrate through the soil and can reach the contamination in-situ. Properties of the nZVI and remediation possibilities depend on methods of production [2]. At the Technical University of Liberec, experiments with iron nanoparticles TODA produced by the company Toda Kogyo Corp. [3] and with the nanoparticles NANOFER, produced by the company NANO IRON s.r.o. [4], are made. During a remedial intervention, transport of the iron nanoparticles is slowed down due to rapid aggregation of them. The rate of aggregation increases with growing concentration of particles in solution and with growing ionic strength of the solution [5]. For the preservation of the transport properties, it is advisable to stabilize the particles. A lot of methods of stabilization were published [6-10]. We simulate the transport of the iron nanoparticles and that is why we examine the interactions among them causing the aggregation. Models of aggregation of small particles were published in many articles (e.g. [11-13]). They are mostly based on the publications [14,15]. However, this generally used model is insufficient for our case. A surface charge established on the surface of particles causes repulsive electrostatic forces between them. The influence on the aggregation into the known aggregation model was implemented [16]. The iron particles corrode in the water, and this process causes change of the surface charge as well as the change of the rate of aggregation [17]. Because the particles are made from iron, they also have magnetic properties, which significantly affects the rate of aggregation [2,18-21]. That is why we want to derive a mathematical model of magnetic forces among particles and to add it into the aggregation model. There is shown a procedure of the derivation of the model in this paper and there is made also an evaluation of the rate of aggregation influenced by magnetic forces here.

The extended model of the aggregation of iron nanoparticles will be included into a solver of particle transport in groundwater. It would allow to simulate the transport of iron nanoparticles and to predict the efficiency of the remedial intervention. That could be useful for the proposal of optimal remedial intervention, which would enable to decontaminate an affected area efficiently and economically.

## Aggregation of Colloids and Small Particles in Groundwater

The particles in groundwater aggregate easily. They create clumps of particles up to the size of several μm [20] that cohere and decrease the possibility of migration of particles through pores of the ground. The aggregation of particles is proven by experiments described in many articles. In [22], particle size, size distribution and surface composition were characterized by transmission electron microscopy (TEM), X-ray diffraction, high-resolution X-ray photoelectron spectroscopy, X-ray absorption near edge structure, and acoustic/electroacoustic spectrometry. There are presented micrographs of a single particle and aggregates of iron particles in the article. In [3], characterization of iron nanoparticles using TEM according to methods of its preparation is done. In [20], a type of aggregation according to beginning concentration of iron nanoparticles is studied by dynamic light scattering, optical microscopy, and sedimentation measurements.

The aggregation of the particles is caused by many processes that generally occur during the particle migration. The decrease in the mobility can be formulated by a rate of aggregation that is given by the mass transport coefficients β [m^3^ s^-1^]. It was published in many papers (e.g. [11,15]). The coefficients give a probability *P*_*ij*_ of creation of aggregate from particle *i* and particle *j* together with concentrations *n*_*i*_, *n*_*j*_ of particles *i* and particles *j* (1). Particle *i* means the aggregate created from *i* elementary nanoparticles.

(1) $$P_{ij}=\beta_{ij}n_{i}n_{j},$$

(2) $$\beta_{ij}=\beta_{ij}^{3}+\beta_{ij}^{2}+\beta_{ij}^{1}.$$

The $\beta_{ij}^{3}$ is the mass transport coefficient of aggregation caused by the gravity, $\beta_{ij}^{2}$ is the mass transport coefficient for the velocity gradients, and $\beta_{ij}^{1}$ stands for the mass transport coefficient of the heat fluctuations. The notation is adopted from [11].

The first process that causes the aggregation of particles is sedimentation. Due to the gravitation forces, the particles fall. According to their size, the velocity of the sedimentation is different for the different aggregates. It implies that the particles have a chance to meet the others and aggregate due to the attachment forces. Following [11], the mass transport coefficient for sedimentation is

(3) $$\beta_{ij}^{3}=\frac{\pi g}{72\eta }\;(\varrho_{p}-\varrho ){\left(d_{i}+d_{j}\right)}^{2}|d_{i}^{2}-d_{j}^{2}|,$$

where *g* is gravity acceleration, η is viscosity of medium, *ϱ* is density of medium, *ϱ*_*p*_ is density of aggregating particles, *d*_*i*_ is diameter of particle *i*.

The process that works similarly is water drifting. The flowing water in a pore in soil has a velocity profile and in the middle of the pore the velocity of flowing is largest. Since the particles have different velocities, according to the place where they are in the pore, the particles can meet each other and create the aggregate. Following [11], the mass transport coefficient for the velocity gradients of particles is

(4) $$\beta_{ij}^{2}=\frac{1}{6}G{\left(d_{i}+d_{j}\right)}^{3},$$

where *G* is the average velocity gradient in a pore.

In the case of the small particles, the heat fluctuation of particles has a significant effect on the particle aggregation. Brownian diffusion causes a random movement of the particles and again it facilitates the aggregation. Following [11], the mass transport coefficient for the Brownian diffusion is

(5) $$\beta_{ij}^{1}=\frac{2k_{B}T}{3\eta }\;\frac{{\left(d_{i}+d_{j}\right)}^{2}}{d_{i}d_{j}},$$

where *k*_*B*_ stands for Boltzmann constant and *T* denotes absolute temperature.

A statistical assessment of the importance of the particular processes to the creation of the aggregates was done. The Table 1 shows that for the smallest particles the Brownian diffusion is most considerable. The sedimentation is most significant when the difference between sizes of the aggregates is largest. It is a consequence of the fact that the difference between the velocities of the particles is largest. The velocity gradients depend on the pore size. When the size of pores is small, the aggregation is most influenced by the velocity gradient, if the difference between the particles is large.

**Table 1:** The mass transport coefficients for Brownian diffusion, velocity gradients, and sedimentation, for different sizes of aggregates

*i*	*j*	$\beta_{ij}^{1}$	$\beta_{ij}^{2}$	$\beta_{ij}^{3}$
1	1	1.0 × 10^-17^	2.2 × 10^-20^	0
1	10	1.3 × 10^-17^	8.8 × 10^-20^	5.9 × 10^-22^
1	10^2^	1.9 × 10^-17^	5.0 × 10^-19^	1.0 × 10^-20^
1	10^3^	3.3 × 10^-17^	3.7 × 10^-18^	2.0 × 10^-19^
1	10^4^	6.5 × 10^-17^	3.2 × 10^-17^	3.8 × 10^-18^
1	10^5^	1.3 × 10^-16^	3.0 × 10^-16^	7.9 × 10^-17^
1	10^6^	2.8 × 10^-16^	3.0 × 10^-15^	1.7 × 10^-15^
1	10^7^	6.0 × 10^-16^	2.8 × 10^-14^	3.5 × 10^-14^
10	10	1.1 × 10^-17^	2.2 × 10^-19^	0
10^2^	10^2^	1.3 × 10^-17^	8.8 × 10^-19^	1.2 × 10^-20^
10^3^	10^3^	1.1 × 10^-17^	2.2 × 10^-17^	5.9 × 10^-18^
10^4^	10^4^	1.3 × 10^-17^	8.8 × 10^-17^	0

The mass transport coefficient for the velocity gradients is quantified for the case with a small size of pores and a large flux (flux = 3.67 × 10^-4^ ms^-1^, porosity = 0.39, velocity gradient *G* = 50 s^-1^). In the other cases, the mass transport coefficient would be much smaller than the others.

## Electrostatic Forces Among Iron Nanoparticles

A mathematical model of aggregation for the case of iron particles was compiled. The sedimentation, velocity gradients, and Brownian diffusion are not sufficient for the description of the process aggregation. The surface of iron particles oxidizes. The ions on the surface attract ions from the electrolyte and the electric double layer arises. Therefore, the influence of the electrostatic forces was added in the mass transport coefficients. The detailed derivation of it is published in [16,23].

For sedimentation, the mass transport coefficient $\beta_{ij}^{3el}$ has the following form:

(6) $$\beta_{ij}^{3el}=\beta_{ij}^{3}-\frac{\pi d_{i}^{2}d_{j}^{2}\sigma_{i}\sigma_{j}}{12\eta \varepsilon_{0}}\left|\frac{1}{d_{i}}-\frac{1}{d_{j}}\right|,$$

where σ_*i*_ and σ_*j*_ stand for surface charge on particle *i* and particle *j*, respectively, *ε*_0_ is permittivity of the medium. If the term that reduces the mass transport coefficient is greater than the mass transport coefficient without the influence of electrostatic forces $\beta_{ij}^{3}$, the probability of collision of particles *i* and *j* should be equal to zero. That is why

(7) $${\tilde{\beta }}_{ij}^{3el}=\max(0,\beta_{ij}^{3el}).$$

For the velocity gradients, the mass transport coefficient $\beta_{ij}^{2el}$ is equal to

(8) $$\beta_{ij}^{2el}=\beta_{ij}^{2}-\frac{\pi d_{i}^{2}d_{j}^{2}\sigma_{i}\sigma_{j}}{12\eta \varepsilon_{0}}\left|\frac{1}{d_{i}}+\frac{1}{d_{j}}\right|,$$

(9) $${\tilde{\beta }}_{ij}^{2el}=\max(0,\beta_{ij}^{2el}).$$

And finally, the Brownian diffusion gives the mass transport coefficient $\beta_{ij}^{1el}$:

(10) $$\beta_{ij}^{1el}=\beta_{ij}^{1}-\frac{\pi d_{i}^{2}d_{j}^{2}\sigma_{i}\sigma_{j}}{3\eta \varepsilon_{0}(d_{i}+d_{j})},$$

(11) $${\tilde{\beta }}_{ij}^{1el}=\max(0,\beta_{ij}^{1el}).$$

The probability of particle collision decreases quadratically with the quantum of the surface charge of particles. The total probability of the aggregation of a particle *i* with a particle *j* is then

(12) $$P_{ij}=({\tilde{\beta }}_{ij}^{3el}+{\tilde{\beta }}_{ij}^{2el}+{\tilde{\beta }}_{ij}^{1el})n_{i}n_{j}.$$

**Figure 1:**
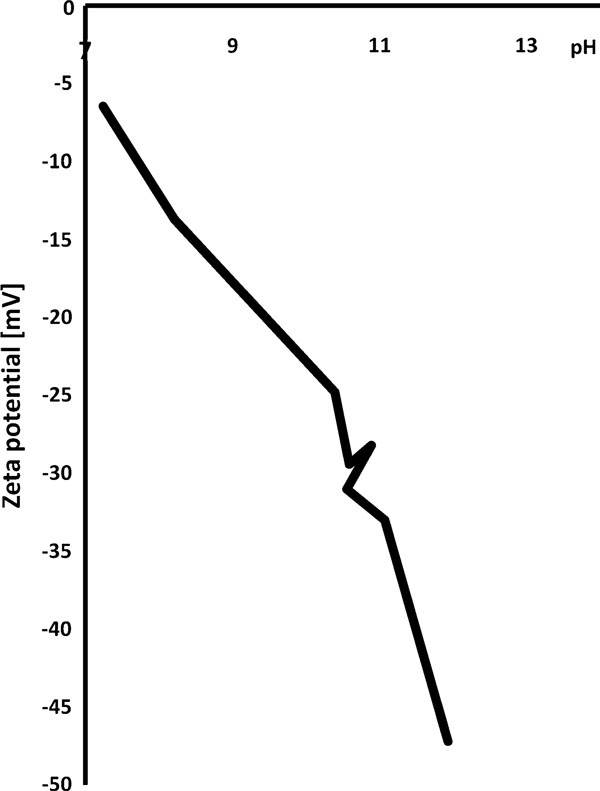
**Dependence of ζ potential of nZVI on pH measured with Malvern ZetaSizer**.

Values of mass transport coefficients for aggregates with the sizes between 50 nm and 5 μm are computed. The surface charge depends on ζ potential (see e.g. [24]). ζ potential depends on pH of the water. The measured results of this dependence acquired using the Malvern ZetaSizer are shown in the Figure 1. Zero-valent iron provides alkaline reaction in water, so the measurement was done for higher pH values only.

The statistical assessment of the importance of the electrostatic forces to the creation of the aggregates was done in [16,23]. The results are that the mass transport coefficient for Brownian diffusion is limited by the electrostatic forces mostly for large aggregates. The mass transport coefficient for the velocity gradients is not limited by the surface charge for measured ζ potential. The ζ potential would have to be minimally ten times larger to affect the rate of aggregation. The mass transport coefficient for sedimentation is limited by the surface charge mostly for the small particles.

## Magnetic Field of Iron Particles

**Figure 2:**
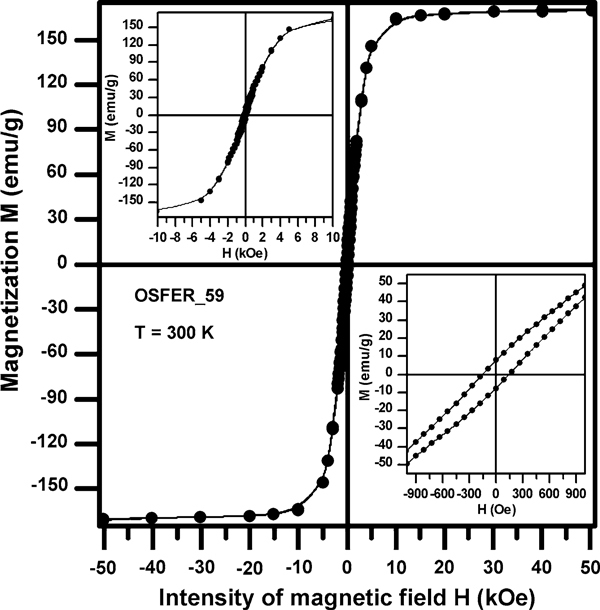
**Hysteresis loop of the zero-valent iron nanoparticles for temperature of 300 K measured with magnetometer MPMS XL by Jiří Tuček at the Palacký University Olomouc**.

The iron particles NANOFER that are used for the remedial intervention were measured by magnetometer MPMS XL, an equipment based on the SQUID effect (Superconducting quantum interference device), owned by the Palacký University Olomouc, Czech Republic. The iron particles are ferromagnetic, a hysteresis loop of the iron particles measured by SQUID is in Figure 2. That is the reason why it is necessary to include the influence of the magnetic forces among particles to the model of aggregation of the particles. However, it is a very complicated process which cannot be described analytically. That is why only the description of the magnetic forces between two particles is shown in this paper. Although it is not a sufficient model, it can be used for determination of something what we could call "effective range" of the magnetic forces. Also the limits of the sizes of the magnetic forces can be determined. It can be used for the assessment of the aggregation of the particles depending on their concentration.

According to [25], the electromagnetic potential in the point **r** near a permanent magnet is equal to

(13) $$\phi (r)=\int_{V}\frac{MR}{R^{3}}dV,$$

where the vector **M** is the vector of magnetic polarization at the point *dV*, the vector **R** is the difference between the source of magnetic field *dV* and the point **r**, *R* is the length of **R**.

Intensity of the magnetic field **H** can be subsequently computed as

(14) H(r)=−grad(ϕ(r)).

Finally, the magnetic force between the source of the intensity of magnetic field **H** and a permanent magnet with the vector of polarization **M**_0_ at the point **r** is equal to

(15) $$F(r)=-\int_{V}\left(M_{0}\cdot grad)H(r\right)dV.$$

### Magnetic Forces Between Two Spherical Iron Nanoparticles

The scalar potential of the magnetic field around one homogeneous spherical iron nanoparticle with radius *a* located at the point [0, 0, 0] was determined:

(16) $$\phi (r)=M\int_{0}^{2\pi }\int_{0}^{\pi }\int_{0}^{a}\frac{\left(x_{3}-r^{\prime }\text{cos}(\theta ))r^{\prime 2}\text{sin}(\theta \right)}{\sqrt[{3}]{{\left(x_{1}^{2}+x_{2}^{2}+x_{3}^{2}-r^{\prime 2}\right)}^{2}}}dr^{\prime }d\theta d\varphi ,$$

where *a* is the radius of the nanoparticle and [*x*_1_, *x*_2_, *x*_3_] are the coordinates of the point **r**. The direction of the vector of polarization **M** is set to the direction *x*_3_, *M* is the magnitude of the vector **M**.

After integration, the magnetic potential around a ferromagnetic sphere is obtained:

(17) $$\phi (r)=4M\frac{\pi x_{3}\left(a-\arctan\left(\frac{a}{\sqrt{x_{1}^{2}+x_{2}^{2}+x_{3}^{2}-a^{2}}}\right)\sqrt{x_{1}^{2}+x_{2}^{2}+x_{3}^{2}-a^{2}}\right)}{\sqrt{x_{1}^{2}+x_{2}^{2}+x_{3}^{2}-a^{2}}}.$$

According to (14), the components of the vector of intensity of the magnetic field around a spherical ferromagnetic particle is

(18) Hi(r)=δi3[−4πC(r)(a−arctan(aC(r))C(r))]−4πx3C(r)(axir·r−xiC(r)arctan(aC(r)))+4πx3xiC(r)3(a−arctan(aC(r))C(r)),

where δ_*i*3_ stands for Kronecker delta and *i* = 1, 2, 3. The symbol *C*(**r**) replaces

(19) $$C(r)=\sqrt{x_{1}^{2}+x_{2}^{2}+x_{3}^{2}-a^{2}}.$$

**Figure 3:**
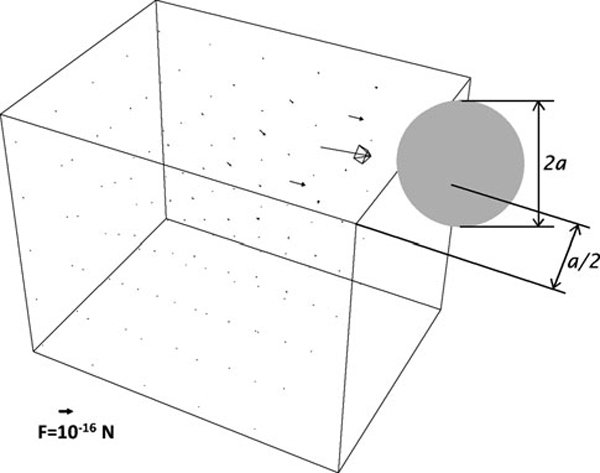
**Visualization of the vector field of the magnetic forces between two spherical particles of nZVI, using software Mathematica 5, copyrighted by Wolfram Research, Inc**. One nanoparticle is in an arbitrary point near a nanoparticle with radius *a* which is touching the center of the upper right side of the figure.

The derived formula of the size of the magnetic forces between two iron nanoparticles is very extensive; hence, it is not presented here. Though, an example of the numerical result is shown. In Figure 3, there is the visualisation of a part of the vector field of the magnetic forces between two nanoparticles. First nanoparticle is in an arbitrary point near second nanoparticle with radius *a* which is touching the center of the upper right side of the figure. The figure is created by the software Mathematica 5, copyrighted by Wolfram Research, Inc.

### Magnetic Field Around an Aggregate

The aggregate of iron nanoparticles is in fact a clump of many permanent magnets. It is impossible to establish an analytical model of interaction of two such aggregates. To analyze statistically the influence of the magnetic forces on aggregation of two nanoparticle aggregates, a script for the examination of the worst possibility (the largest forces) and the averaged possibility of influencing the aggregation by the magnetic forces was written down.

The statistical model of the aggregate is made so that the volume of the aggregate is filled by uniformly distributed nanoparticles (small homogeneous magnets) with randomly uniformly distributed direction of the magnetic polarization. The magnitude of the magnetic polarization of all nanoparticles is the same. The magnetic potential of the aggregate is then the superposition of magnetic potentials of all nanoparticles creating the aggregate:

(20) $$\phi (r)=\sum_{i}\phi_{i}(r-r_{i}),$$

where φ_*i*_ is potential of magnetic field of the nanoparticle located in the point **r**_*i*_.

The influence of magnetic forces in comparison with the gravitational forces is investigated. It could be compared also with other affecting forces but the gravitation force was chosen for the reason of small number of variables. If one aggregate is in a fixed position and another one is located somewhere vertically under it, there should be a unique distance ("effective range") between them so that if they are closer than it, the lower aggregate would attach to higher one by magnetic forces. If they are more distant, they would sediment separately. The distance is measured between the surfaces of particles.

**Figure 4:**
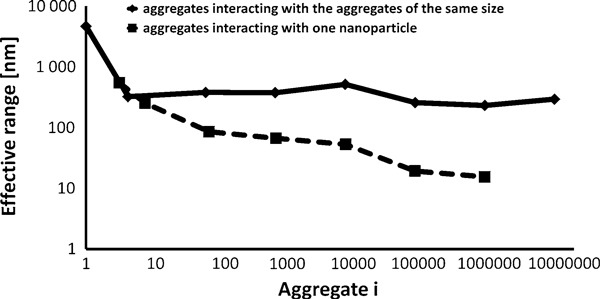
**Effective range of the magnetic forces of chosen aggregates**. The *dashed line* characterizes the effective ranges for the interaction of aggregates interacting with a single nanoparticle. The solid line characterizes the effective ranges for the interaction of two aggregates of the same size. The graph is plotted for the magnetic polarization 170 emu g^-1^.

In Figure 4, the dashed line characterizes the effective ranges for interaction of chosen aggregates interacting with a single nanoparticle. The solid line in the graph characterizes the effective range for the interaction of two aggregates of the same size. The absolute value of the magnetic force and consequently also effective range quadratically depends on the magnitude of the magnetic polarization. The graph is plotted for the magnetic polarization 170 emu g^-1^. More important information than absolute values is the trends of the lines.

According to the results from the Table 1 in the Section 2, sedimentation influences the aggregation mostly when the difference between sizes of the two aggregates is large. Consequently, the dashed line in the Figure 4 gives a good information about the influence of magnetic forces on aggregation of aggregates of different sizes. The solid line comparing the influence of magnetic and gravitational forces between two similar aggregates does not include the real information about the influence of magnetic forces because another force than gravitational governs the aggregation process in such a case.

On the basis of this result, it is obvious that the magnetic forces among particles have significant effect on the rate of aggregation of the particles. On the basis of the effective range, concentration of particles uniformly dispersed in solution can be computed. According to distances between particles, the concentration would have to be very small (about 15 mg l^-1^) to be possible to neglect the influence of the magnetic forces.

## Conclusion

The influence of the magnetic forces among iron particles on the rate of aggregation in terms of the "effective range" was assessed. The effective range is a distance in which the magnetic forces outweigh the gravitation force that causes the aggregation. To assess the magnetic field around more interacting aggregates, it should be made a model using the Finite Element Method (FEM) or another numerical method. The next steps in the studying of nanoparticle aggregation due to magnetic forces can be the evaluation of the effective range in comparison with other forces indicated in this paper and/or building a FEM model of nZVI aggregate.
